# Is “bioinformatics” dead?

**DOI:** 10.1371/journal.pbio.3001165

**Published:** 2021-03-18

**Authors:** Philip E. Bourne

**Affiliations:** University of Virginia, Charlottesville, Virginia, United States of America

## Abstract

Why would a computational biologist with 40 years of research experience say bioinformatics is dead? The short answer is, in being the Founding Dean of a new School of Data Science, what we do suddenly looks different.

Now that I have your attention, clearly, bioinformatics as a field is very much alive. The name, however, no longer applies to what we actually do in the field. It is not what forward-thinking scientists should be calling themselves in this era of the fourth paradigm of data science [1], where data sharing lies at the core of biology. If you’re asking why anyone should care, let me explain.

But first, let me acknowledge Florian Markowetz, who started the discussion [2]. As NIH Associate Director for Data Science, I made similar arguments to the Advisory Committee to the NIH Director in 2014. I argued that until the 1980s to 1990s, computation was a complex tool in the hands of a few. The human genome project changed all that. Experiment and computation were synergistic and the promise of bioinformatics—which could not only describe and maintain the massive volume of digital data being generated but also provide the tools needed to assemble and make sense of 3 billion nucleotides—claimed the limelight. The euphoria around what bioinformatics could accomplish was so great that in the early 2000s the private sector snatched up a significant fraction of the computational practitioners to capitalize on the era of human genomics. The initial excitement faded several years later, when bioinformatics could not deliver in the short product cycles that the industry demanded, and bioinformaticians were regarded as mere service providers to experimentally driven research.

Still, its promise could not be denied. A new generation of practitioners emerged who were as adept in silico as they were in vivo and/or in vitro. Bioinformatics spread its wings as computational biology and then systems biology, which is roughly where we are today. In 2014, I predicted that by 2020, computation would be steering the biomedical ship, experiment would increasingly be used to confirm predictive models, and causation would increasingly be determined from digital data previously collected by others or by bioinformatically guided laboratory robotics that maximized experimental insights.

My predictions about timing were off, but not about outcome. The role of what I’ll call digitally based causation and predictive analytics is assured. Thus, how we describe our field, to truly express what we do, is off. What’s driven the changes? The same thing that spawned bioinformatics: digital data. But now there is much more of it, and it’s not just DNA sequences but data at all scales, from molecules to populations, and it’s being collected (high-throughput methods) and generated (via simulations and modeling) at unprecedented rates. Add to that hardware and software architectures that support a variety of increasingly robust machine learning techniques and you see where we are headed.

Data, or as we now refer to it, “big data,” drive change. It’s embodied in the field of data science. If you call yourself a “bioinformatician,” you may be thinking, “what’s the big deal, I’ve been doing data science all along.” But I would argue that something fundamentally different is going on.

My career spans a large part of the third paradigm of computation [1], and the changes happening now in academia are unprecedented. All disciplines are undergoing transformations akin to genomics but in a tenth of the time. I say this from the vantage point of developing a School of Data Science that works with all academic disciplines and private sector verticals, from health to finance. Every field is training bioinformaticians to open up new analytical branches of their traditionally empirical fields. This will influence what we do in biomedicine: what tools we use, what data we analyze, how our culture is impacted, and even how we think about ourselves.

Bioinformatics researchers may think we have a unique “in” to the fourth paradigm, derived from a sense of being first as well as limited exposure to other domains that’s reinforced by the siloed walls of academia. Data science in academic institutions across the world is changing that view. Wherever a data science initiative lives within an academic institution, it is breaking down those silos, simply by being interdisciplinary. Data science—part computer science, part statistics, part information science, and part applied math—with its unique blend of methodologies and scientific cultures—doesn’t acquire meaning until put to practical use. Add to that the influence of the private sector, think DeepMind [3], and you have something new and groundbreaking. As an example, we describe ourselves as a “school without walls”*—*the “school” gives us autonomy in an academic institution, and the “without walls” means we don’t own anything but contribute to everything (or at least aim to).

This arrangement creates an institutional nexus to exchange best practices between disciplines in a way that did not exist previously. It recognizes that new developments relevant to biomedical research will happen far beyond the walls of biomedicine, and the most successful biomedical researchers will embrace these opportunities first. There is nothing fundamentally new here. Recall that hidden Markov models developed in the 1960s for signal processing found their way into bioinformatics in the 1980s, and ontological research with roots in artificial intelligence (AI) influenced the development of the gene ontology. What is fundamentally new is the speed of adoption and the locus of new opportunities.

How data science can be a catalyst of change depends on how you define data science (see Box 1). But its potential as a catalyst has never been greater given the unprecedented depth (coarse to fine grained), breadth (multimodal/multi-type), and sheer volume of data. The promise of multiscale modeling—working from molecules to populations in a single study, with the ability to detect and map causal inferences across scales—is a strong driving force, as it opens the door to truly integrative theories of biological systems. To get there, we need a quantum leap in training, with mentors versed in data types across scales and methods either derived from or at least applied in different fields. We need to be more aware than ever of developments that may be far outside our discipline that fall under the broad topic of data science. In short, we need to become biomedical data scientists.

That name better reflects what we do, or certainly what the students we are training today will do. It really doesn’t matter what you call it. Just do it.

**Box 1. What is data science? The 4+1 model.**

Within our School of Data Science, we have given a great deal of thought to this question [3], focusing not so much on the definition itself, but rather on how we embody the meaning and culture of that definition in all aspects of teaching, research, and service to the community. As such, we have arrived at the 4+1 model of data science.

The “4” refers to systems, design, analysis, and value, and the “1” to all the disciplines/domains to which these fundamentals are applied. Bioinformatics is only one of the many disciplines/domains.

Each of the “4” brings forth the influence of the “1,” through the data, methods, protocols, culture, etc. of those fields. Synergistically, the “4” then influences the “1.” Three examples of this synergy include the influence of available environmental data on epigenetics, the influence of social media networks on our understanding of biological networks, and advances in the use of natural language processing in the digital humanities influencing developments in biocuration from the literature.

Systems implies high performance computing, cloud environments, high-throughput systems, workflow systems, benchmarking analyses, cybersecurity, and the like, all tuned to the needs of big data.

Design concerns the relationship between data and computers and humans at all stages of the data life cycle, from ingestion through to visualization and other forms of dissemination.

Analysis, mistakenly considered as all of data science, is only one important part and comprises techniques seeking both causality and prediction such as deep learning, data mining, and natural language processing.

Value implies, at its extremes, the tension between the value an analysis brings versus the unintended negative consequences that can result and how to strike the right balance.
